# Feature derivation and classification of auditory-motor coupling dynamics in healthy and neurologically impaired adults

**DOI:** 10.1371/journal.pone.0315607

**Published:** 2024-12-16

**Authors:** Muhammad Bergas Nur Fayyad, Joeri R. Verbiest, Anna Ivanova, Mario Manto, Lousin Moumdjian

**Affiliations:** 1 IQ Health Department, Section Biostatistics, Radboud University Medical Center, Nijmegen, Netherlands; 2 Faculty of Rehabilitation Sciences, REVAL Rehabilitation Research Center, Hasselt University, Hasselt, Belgium; 3 Biomedical Research Institute (BIOMED), Hasselt University, Diepenbeek, Belgium; 4 Data Science Institute (DSI), Hasselt University, Diepenbeek, Belgium; 5 University Multiple Sclerosis Center (UMSC), Hasselt-Pelt, Belgium; 6 Center for Statistics (CenStat), Hasselt University, Diepenbeek, Belgium; 7 Service de Neurologie, CHU-Charleroi, Charleroi, Belgium; 8 Service des Neurosciences, University of Mons, Mons, Belgium; 9 IPEM Institute for Systematic Musicology, Ghent University, Ghent, Belgium; Prince Sattam bin Abdulaziz University, SAUDI ARABIA

## Abstract

The use of auditory stimuli in rehabilitation to target walking has been evidenced in persons with neurological conditions. The methodologies focus on the synchronisation of persons’ steps to auditory stimuli showing that the type of stimuli and tempi significantly affect the synchronisation. However, the dynamic of the interaction over time between the motor system and the auditory stimuli, i.e., when steps are aligned (termed as locking) and not aligned (termed as unlocking) to the beat of the stimuli, remains unclear. Quantifying these dynamics would assist in the development of personalised rehabilitation. Nevertheless, it is currently challenging given the variability of responses per individual over time. We propose a methodological solution to quantify the dynamics of the step-to-beat coupling over time within an experimental paradigm where healthy (n = 7) and neurological impaired (n = 6) participants walk three minutes to music and metronomes at various tempi. We applied window partitioning within the time series to account for the changing pattern. To classify data into locked and unlocked events, features of fluctuation and trend were derived on which two statistical tests (circular statistical test and slope test) were done, respectively. Based on the ground truth, the performance of our proposed method yielded high accuracy (91%), precision (90%) and recall (97%). The standard deviation of the inter-step intervals was then modelled across the label and experimental factors. The proposed method is suitable for quantifying fine-grained observation of the dynamics of auditory-motor coupling in adult healthy and neurological impaired participants, with the potential of designing personalised rehabilitation.

## Introduction

The use of auditory stimuli in rehabilitation to target walking has been evidenced in persons with neurological conditions, for example, in persons with Parkinson’s disease [1], stroke [2] and multiple sclerosis [3]. These methodologies are based on sensory-motor synchronisation, where persons are requested to synchronise their steps in time to the beats of music or metronomes. Changes in walking parameters (such as spatiotemporal parameters, e.g., cadence, speed, and stride length) in these studies evaluate the effect of the application of these techniques on the study participants. On the other hand, measuring the synchronisation consistency and accuracy of the step relative to the beat when engaging in such a task also provides abundant information in addition to the changes and dynamics of the walking itself [4].

In a study on neurological patients, our research group previously showed that synchronisation to auditory stimuli significantly differs when walking to music compared to metronomes for different auditory tempi [3,5]. The results obtained per trial of three minutes were summarised into one average value per trial—as commonly done to report on central tendencies in statistical reporting [6]. Albeit these results are comprehensive and provide valuable clinical insights, they still fail to report on the dynamics of the interaction over time. To elaborate, that is the investigation of the dynamics of the interaction occurring between the motor system (intrinsic oscillator) and the auditory stimuli (and extrinsic oscillator) when engaging sensorimotor synchronisation. This study aims to address this gap by quantifying the dynamics of auditory-motor interactions over time, rather than solely focusing on statistical metrics such as averages. Understanding these dynamical processes over time can advance both fundamental and clinical applications. For instance, a clinician, with the help of assistive technologies, can intervene and adjust the components of the auditory-motor paradigm in real time when suboptimal dynamics are detected, enabling in this way a more personalised and effective intervention.

However, quantifying these dynamics over time across participants and trials is challenging given the differences, not only across participants but also within participants [7]. We propose a methodology to tackle this challenge during a task when persons are instructed to synchronise their steps during walking to auditory stimuli (music and metronomes). The relative phase angle (rPA), a measure of an individual’s step and the closest beat in the auditory stimuli [4], is an outcome to quantify step-to-beat synchronisation. If one applies a classification logic [8], two possibilities arise: a) participants are locked in phase (i.e., synchronising: their steps are aligned with the beat), and b) participants are not locked in phase (i.e., not synchronising: their steps are not aligned with the beat).

Given the challenges mentioned above, we proposed a methodological framework that takes into account the time domain of the signal. It also avoids specifying arbitrary thresholds for mean and variance in the classification of periods of locking and unlocking. The method includes partitioning the trial into time windows and classifying each window into locked and unlocked states by a systematic statistical evaluation. Afterwards, the time series specific to the walking (gait interval variability) can be merged into the partitioned windows. We hypothesised that this methodological framework will enable the investigation of auditory-motor coupling dynamics over time thereby allowing answering the following research questions. Does locking or unlocking over time within a trial affect the variability of inter-step interval across stimuli and tempi. Answers to such questions can guide the rehabilitation community in developing targeted and individualised rehabilitation plans. As well as contribute to the advancement of assistive technologies, enabling the implementation of personalised rehabilitation strategies that optimise auditory-motor dynamics. We applied this methodology in adult populations with and without neurological impairments.

## Methods

### Experimental procedure and data

This experiment is part of a larger-scale project, medical ethical approval (B1152021000003) and clinical trial registration (NCT04887753) investigating sensorimotor synchronisation in persons with cerebellar impairments. The recruitment period for the large-scale project started in 29^th^ of April 2021, and ended on the 3^rd^ of May 2024. Recruited participants provided a written consent for study participation by signing the informed consent documentation approved by the medical ethical committee. The following inclusion criteria was used for cerebellar-impaired persons: the presence of a cerebellar impairment diagnosed by a neurologist evidence from MRI imaging (presence of a lesion and/or degeneration), or a minimum score of 1 on the Scale of Assessment and Rating of Ataxia. Participants were excluded if they exhibited cognitive impairment impeding understating the instructions, uncorrected hearing impairment, beat amusia, or pregnancy.

Each participant was equipped with an interactive music player called the D-jogger [9]. The D-jogger consists of headphones (Sennheiser RS 127–8 headphones), inertial measurement unit sensors (NGIMU,x-io technologies limited) strapped to the ankles, and a laptop ((Dell Latitude laptop, Core i5-1145, Windows 10 Pro, ASIO low-latency soundcard).) with a custom-made software. The D-jogger provided auditory stimuli at any given tempo by changing the frequency of the musical beats and metronome ticks and logged all auditory and gait data for calculating outcome measures of gait-music synchronisation discussed in the following paragraphs.

The D-jogger technology has been applied in previous studies to provide and quantify step-beat synchronisation during walking and/or running on adult healthy controls [9–11] and neurological populations [3,5,12–14] as well as in children with developmental coordination disorder [15]. The NGIMU sensors have been validated with gold standard gait analysis methods [16].

Each participant was asked to walk to different trials of 3 minutes each. The trials consisted of two blocks of stimuli (music and metronomes) at seven tempi: -12%, -8%, -4%, 0, +4%, +8%, and +12% of baseline comfortable cadence. Thus, each participant underwent 14 different trials.

### Outcome measures

**Fig 1:**
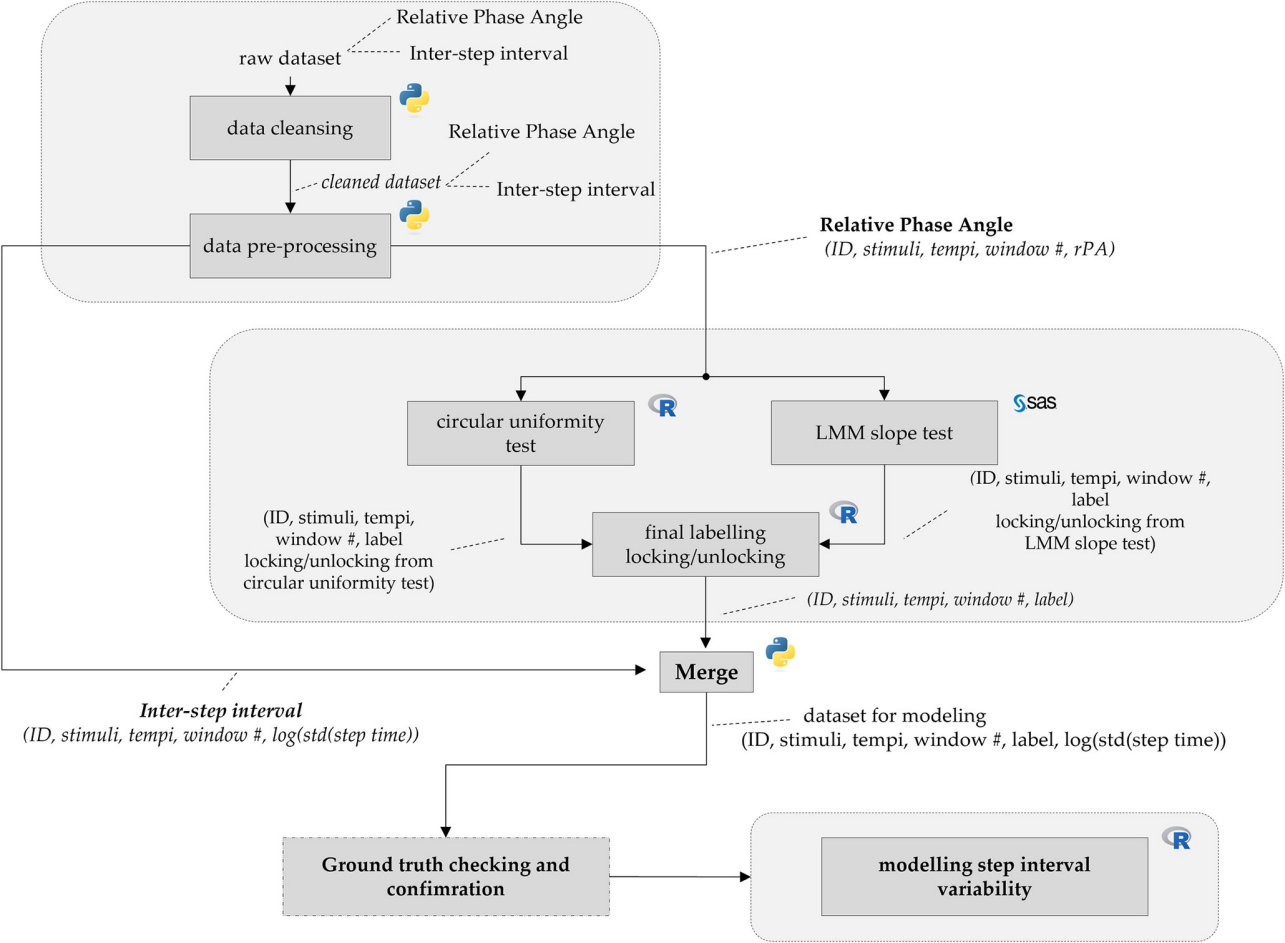
Pipeline of the analysis.

As seen in Fig 1, two time series measurements are processed: the inter-step interval and relative phase angle. The measurements were derived for each participant and each trial.

#### A. The inter-step interval

The inter-step interval describes the participant’s walking pattern and was derived from the inertial measurement unit sensors (NGIMU, UK) recordings of the step-time. Step_*j*_ denotes the time at which the *j*-th step occurs. We define the time variable *t*_*j*_ which denotes the timing of the *j*-th step (*t*_*j*_ = Step_*j*_). The inter-step interval *δ*_*j*_ at time *t*_*j*_ is calculated by subtracting the (*j*+1)-th step with the *j*-th step.


$$\delta_{j}={\mathrm{Step}}_{j+1}-{\mathrm{Step}}_{j},\;j=1,2,\ldots ,J.$$

Note that the number of observations of the inter-step interval is *J*−1 since there is no step occurring after the last step Step_*j*_.

#### B. Relative phase angle (rPA)

The relative phase angle (rPA), measured in degrees, describes the participant’s interaction with the auditory stimuli. It quantifies the difference between the step and the closest auditory beat (i.e., measures the timing of the footfall relative to the closest beat), ranging from -180 to +180. This is either a negative (footfall before the beat) or a positive (footfall after the beat) angle. The rPA is calculated as follows:

$$r_{j}=360*\left(\frac{{\mathrm{Step}}_{j}-{\mathrm{Beat}}_{j}}{{\mathrm{Beat}}_{j+1}-{\mathrm{Beat}}_{j}}\right),\;j=1,2,\ldots ,J,$$

where Beat_*j*_ is the time of the beat prior to Step_*j*_. One can calculate the average rPA using circular statistics [17].

### Data pre-processing

As shown in Fig 1, each outcome measure underwent a separate processing step. We carefully examined the time series of the inter-step intervals to identify any technical sensor glitches or faulty observations. To accomplish this, we analysed data from the inertial sensors by visually assessing the gyroscopic sensor data to detect distinct points corresponding to gait events such as mid-swing and initial contact. A very large and very small inter-step interval—such as three seconds and 0.1 second—are likely to be outliers and invalid. Generally, there is no fixed threshold to determine whether the inter-step interval is invalid. For this reason, observations larger than (median +3*sd*) or smaller than (median −3*sd*), where *sd* denotes the standard deviation, were considered outliers and removed from the analysis [18].

Next, the inter-step interval and the rPA time series were verified and adjusted to contain 180 seconds of recording by removing the observations above the 180-second mark. Next, the time series were divided into nine windows, each spanning 20 seconds. On average, these 20-second intervals included 20–30 steps.

#### rPA time series

In this section, we describe the methodological solution where we process the quantification of synchronisation over time. The method allows the capture of the dynamic behaviour of synchronisation that varies across and within participants and trials. The proposed solution was to label each partitioned window within the time series with two labels: locked (good synchronisation occurring) or unlocked (i.e., synchronisation not occurring). To perform the labelling, the fluctuation and the trend of rPA are evaluated in two different manners separately with an interchangeable order. The fluctuation and the trend of rPA evaluation give label results after which we combine both with an "if else" logic for the final labels. Below, we provide the details:

*a. Evaluation of fluctuation (circular uniformity test)*

In evaluating the fluctuation, one may consider calculating the variability (e.g., standard deviation) of the rPA and determine that high variability indicates an unlocking phase and vice versa. However, this approach requires an arbitrary threshold of variability, which cannot be specified by the highly variable individual observations of the participants across trials.

Therefore, circular statistical test [17] is applied since rPA can be seen and analysed as circular data. Circular statistics deals with data that has no true zero where the beginning and the end of the measurement coincide. The distribution of data is different from the linear case. An example of circular data is the von Mises distribution which is comparable to the normal distribution in the linear case. We refer to Jammalamadaka & SenGupta (2001) [19] for further explanation on circular statistics.

The rPA with slight fluctuation is equivalent to circular data concentrated towards a mean direction. On the other hand, high fluctuation implies that the observations are spread over a circle. Thus, the circular uniformity test on the RPA was performed to evaluate the fluctuation. The null hypothesis is that the data is uniformly distributed, which implies high fluctuation, and the alternative is that the data is concentrated without any preferred mean direction, which implies low fluctuation. Therefore, if the null hypothesis is rejected, we label the window as the locking phase.

*b. Evaluation of trend (window-specific slope test)*

One approach to evaluate the trend is to obtain the slope of the rPA over time in each window. Since no general threshold existed, an average trend (slope) over all windows in each trial had to be calculated. We approached this evaluation using a linear mixed model (LMM). LMM is often used in longitudinal studies involving repeated measurements that feature two sources of variation, i.e., between and within individuals. LMM accounts for both sources of variation by the inclusion of a random effect. We refer to Verbeke & Molenberghs (2009) [20] for a detailed explanation on LMM.

The model was fitted with the rPA against the time relative to the starting point of the window and including window-specific random intercept and slope. Then, the estimate and the standard error for every window-specific deviation from the overall slope was obtained using best linear unbiased predictor (BLUP) [21]. Finally, based on the estimate and the standard error, we tested the significance of each window-specific deviation from the overall slope. We labelled the window as the unlocking phase in each trial if the window-specific slope deviated significantly from the overall slope.

*c. Final labelling of each partitioned window*

We combined the results of labels from the above evaluations: a. the circular uniformity test and b. the window-specific slope test.

- A final label of ’locked’ was given to the window where both evaluations indicated ’locked.’. This indicated a stable rPA derived from low fluctuation and flat trend features.
- Otherwise, the window was given a final label of ’unlocked’. This indicated unstable rPA derived from high fluctuation and non-flat (slope) trend features.

Ground truth verification of the final attributed labels was conducted by computing confusion matrix [22]. The matrix was scored into its four components as follows:

- A true positive (TP) was defined when the label indicated ’locked’ and was coupled with the outcome "correct".
- A true negative (TN) was defined when the label indicated ’unlocked’ and was coupled with the outcome "correct".
- A false positive (FP) was defined when the label indicated ’locked’ and was coupled with the outcome "incorrect".
- A false negative (FN) was defined when the label indicated ’unlocked’ and was coupled with the outcome "incorrect".

After that, the values were counted per outcomes (TP, TN, FP, FN) per partitioned window, and a sum of all was computed; this was used to calculate the recall (TP/TP+FN), precision (TP/TP+FP) and accuracy (TP+TN/Total) of the labels. Two independent reviewers constructed the confusion matrix, and a third reviewer was consulted in case discrepancies arose. The reliability of the ratings was assessed using Cohen’s kappa, which measures the agreement between two initial reviewers. Kappa value of 0.60–0.79 indicates a moderate agreement and kappa value of 0.80–0.90 indicates a strong agreement [23].

### Merging datasets of inter-step interval and RPA

As seen in Fig 1, at this stage, both time series feature nine labelled partitioned windows merged to create a dataset for modelling. Since window partitioning was done in the same manner for both datasets, merging the datasets was essentially transferring the information of the labels from rPA dataset to the inter-step interval dataset. This results in a dataset containing the outcome measure of inter-step interval together with the label ’locked’ or ’unlocked’, as well as the subject identifier (ID) and experimental factors (i.e., stimuli and tempi).

### Modelling inter-step-interval variability

Modelling inter-step interval variability is the final step of the proposed methodology, which leads to applying a statistical model to answer the research question: *“Does locking or unlocking within a trial affect the variability of inter-step interval across stimuli and tempi*.*”* The variability of inter-step interval over time is modelled as a function of time (i.e., window), factors (i.e., stimuli and tempi), and accounting for the different labels (locked/unlocked). To model the variability in steps, we calculated the standard deviation of the inter-step interval in each window. The standard deviation was then used as the response variable for the modelling. Thus, we have a repeated outcome measure of standard deviation for each trial.

Let *δ*_*ijklm*_ denote the inter-step interval for subject *i*, measured at time *t*_*ijk*_ in window *ω*_*k*_ with Tempi_*l*_ and Stimuli_*m*_. For each window, the standard deviation was calculated, given by

$$S_{iklm}=\sqrt{\frac{\sum_{j=1}^{J_{k}}{\left(\delta_{ijklm}-\mu_{iklm}\right)}^{2}}{J_{k}}}$$

where μ_*iklm*_ is the mean of the inter-step interval and *J*_*k*_ is the number of steps in *k*-th window. From the equation, it can be seen that the standard deviation does not include the index *j* and the information of time is no longer carried by t_ijk_. Instead, window ω_*k*_ is the only variable that carries the time information. Now, the standard deviation *S*_*iklm*_ can be seen as a repeated measure over the window ω_*k*_, where the variable window ω_*k*_ is now taken as an ordinal variable. In modelling, the ordinal variable of the window is denoted by Window_*k*_ which takes integer values from 1 to 9.

The standard deviation can be modelled as Gaussian data and non-Gaussian data with gamma distribution since it can take any non-negative value. Therefore, the modelling was done using LMM with possibly transformed data. The variance-covariance matrix is set to be unstructured [21].

### Software

The software used in this study were R version 4.3.2, SAS 9.4, and Python 3. As illustrated in Fig 1, data processing and merging of datasets were conducted in Python. The estimation and the test of the components of random effects using BLUP in LMM was performed in SAS software using PROC MIXED [24] by adding ’solution’ in the ’random’ statement. The circular uniformity test was done in R using the function circ.range(., test = TRUE) in CircStats package [25]. Fitting LMM was done in R using lmer() function in lme4 package [26].

## Results

### 1.1 Participants

In this methodological study, we present data collected on seven healthy controls (age: mean 61 ± 9 years, sex: 5 female, 2 male) and six patients with cerebellar impairments (age: mean 58 ± 17 years, sex: 3 female, 3 male). Table 1 presents the clinical information of each patient, including their diagnosis, year of diagnosis, MRI results, and scores from the Scale for the Assessment and Rating of Ataxia [27]. Assistive devices were not used by the participants.

**Table 1:** Descriptive clinical information of each patient, including diagnosis, year of diagnosis, MRI findings, and scores from the Scale for the Assessment and Rating of Ataxia.

Diagnosis	Year of diagnosis	MRI findings	Gait (0–8)	Stance (0–6)	Sitting (0–4)	Speech disturb-ance (0–6)	Finger chase AVG_L,R (0–4)	Nose-finger test AVG_L,R (0–4)	Fast alternating hand movement AVG_L,R (0–4)	Heel-shin slide AVG_L,R (0–4)
Cerebellar Stroke	2021	Right posterior stroke (posterior inferior cerebellar artery)	1	0	0	0	0	0	0	0
Cerebellar stroke	2021	Multiple lesions of bilateral cerebellar hemisphere	1	0	0	0	0	0.5	0	0.5
Arterior-venous stenosis malformulation	2009	Bilateral posterior cerebellar hemispheric lesions and mild cerebellar atrophy	1	0	0	0	0	0	0	0
Cerebellar stroke	2019	Left hemisphertic cerebellar stroke	2	1	0	1	0.5	0.5	0.5	0.5
Cerebellar stroke	2019	Bilateral posterior inferior cerebellar artery stroke	1	2	0	0	0	0	0	0
Cerebellar stroke	2019	Ischemia in Left posterior cerebellar hemisphere (ASCA)	1	0	0	0	1	0	0	0

### 1.2 Step-interval time series

Processing and removal of invalid observations from the raw data recordings.

**Fig 2:**
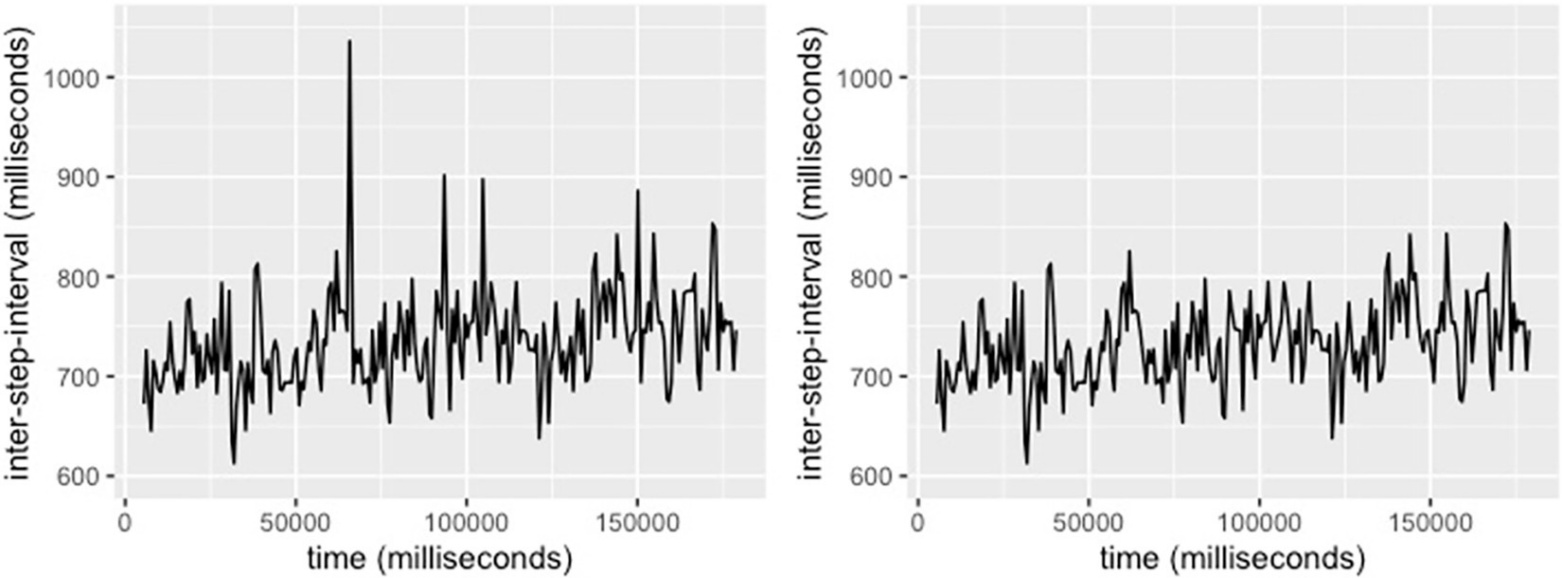
Example of inter-step-interval of a trial, stimuli: Music, tempi: -8% before outlier removal (left) and after outlier removal (right).

As illustrated in Fig 2, invalid observations shown on the left were processed to obtain a clean inter-step-interval time series as shown in the right.

### 1.3 Relative phase angle time series

Labelling of the partitioned windows

*a. Evaluation of fluctuation (circular uniformity test)*

**Fig 3:**
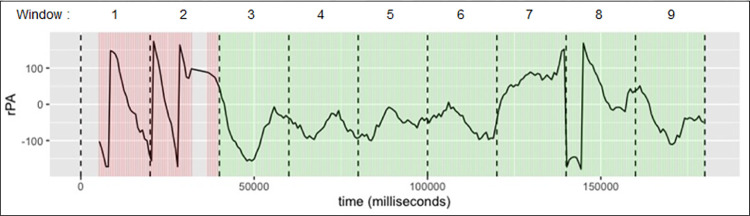
Relative phase angle with colour label for each window based on circular uniformity test: Unlocked (red) and locked (green).

This evaluation was able to catch the phase with high and low fluctuation, although not with full accuracy at all time points. Fig 3, illustrates the time series of rPA during one experimental trial of 180 seconds partitioned into nine windows. The trial has been colour-coded (green and red indicate locked and unlocked window respectively) after undergoing the circular uniformity test. Note that high fluctuation of rPA indicates unlocking phase and, conversely, low fluctuation indicates locking phase. In Fig 3, the rPA in window 1, 2, and 8 fluctuate highly between the values around -150 and 150. It suggests that the steps in these windows were unsynchronised, and thus classified as unlocked. In contrast, the rPA in window 4, 5, and 6 fluctuates between the values around -100 and 0 which suggests that the steps were synchronised and thus classified as locked.

The example illustrates that the unlocked phase was correctly identified in window 1 and 2 and correctly identified ’locked’ label in windows 3 to 6. However, it failed to identify windows 7 and 8 as ’unlocked’.

*b. Evaluation of trend (window-specific slope test)*

**Fig 4:**
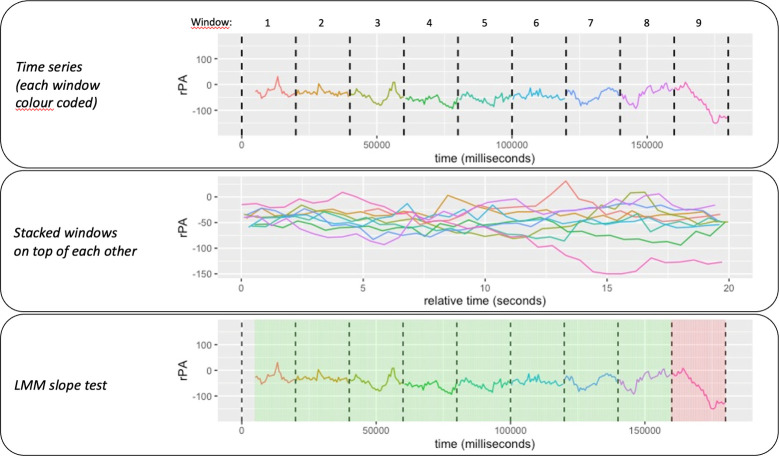
Relative phase angle (rPA) from window 1 to 9 (top panel), rPA in all windows stacked on top of each other (middle panel), and rPA with the locking/unlocking label with green indicating a locking window and red otherwise (bottom panel).

Fig 4, illustrates the time series of rPA during one experimental trial of 180 seconds partitioned into nine windows. The top panel of the figure shows the rPA from window 1 through window 9. The middle panel shows the rPA of nine 20-second windows stacked on each other. The bottom panel shows the label based on the result of LMM slope test. A LMM was fitted with window-specific random intercept and slope on the stacked 20-second windows. Each window-specific slope was tested to determine whether it deviates significantly from the average slope, which is the fixed effect, of the overall 20-second windows. When a significant deviation was absent, the window was labelled ’locked’ (as seen in the first eight windows on the bottom panel of Fig 4). When a deviation was present, the window was labelled ’unlocked’ (as seen by the last window on the bottom panel of Fig 4).

A combination of both evaluations

**Fig 5:**
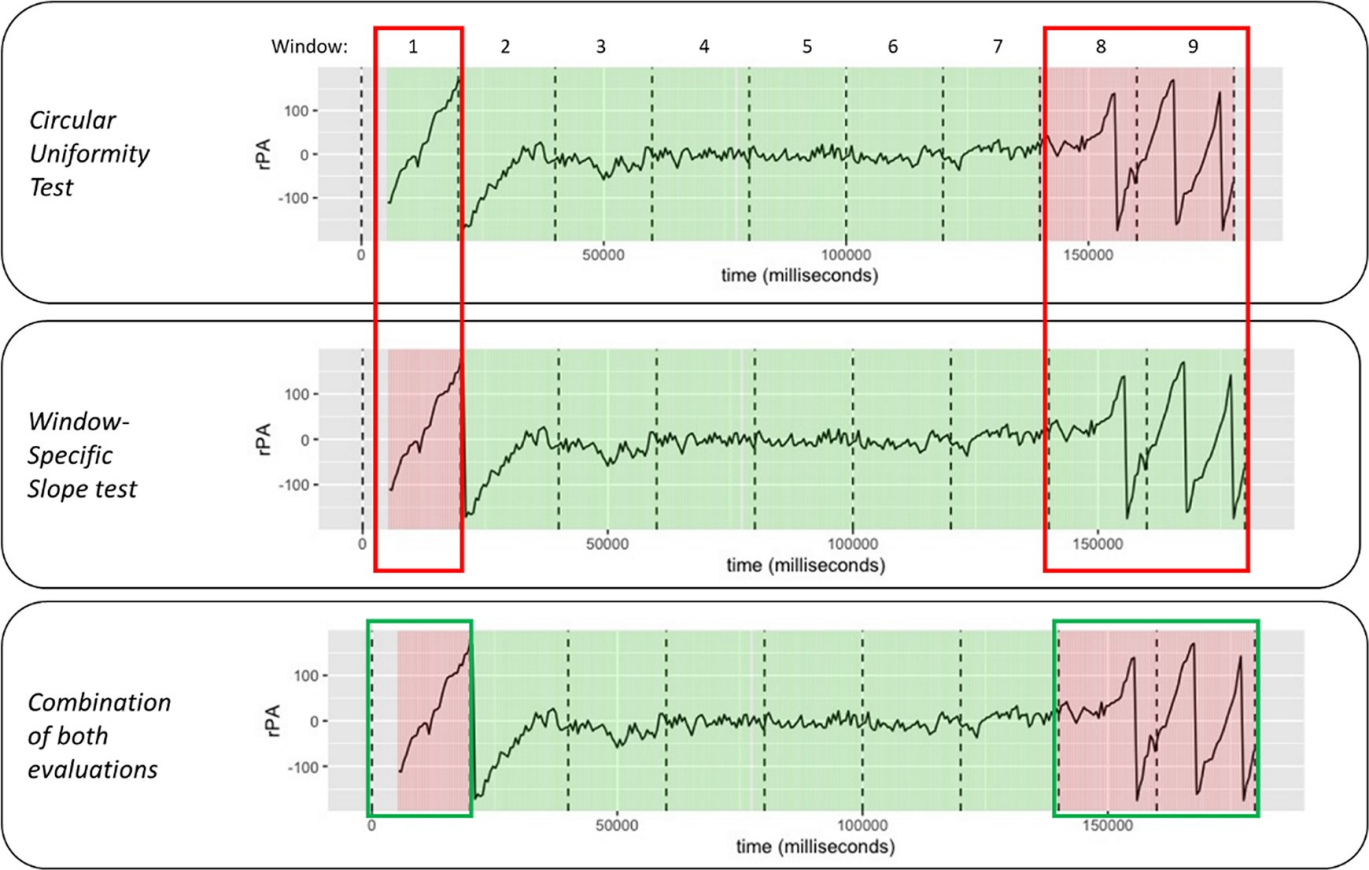
Labelling based on the circular uniformity test (top), slope test (middle), and final label combining both tests (bottom).

Each test captures the locking/unlocking pattern, but not always. With a simple decision rule, the two evaluations were combined. The decision rule was that the window was labelled as unlocked if either of the two evaluations resulted in an unlocked label. Combining both tests improved the performance in capturing the correct labelling. Fig 5 shows a trial where the first window was inaccurately labelled as unlocked with the circular uniformity test, but the slope test was able to provide it with the correct ’unlocked’ label. The opposite case occurred in the last two windows, where the label ’unlocked’ was correctly attributed based on the circular uniformity test but not with the slope test. Nevertheless, the window was attributed to the correct label by combining both pieces of information.

Ground truth verification

The reliability of ratings between the two assessors. The Cohen’s Kappa statistics indicated that the initial two raters demonstrated a moderate agreement (Kappa = 0.744). Out of 1,571 total ratings, the raters disagreed on 82 instances, which were subsequently resolved by the third rater, ensuring consensus.

The results of the confusion matrix. The confusion matrix calculations of the combined methodology, accounted for an accuracy of 91%, a precision of 90% and a recall of 97%. Thus, the proposed methodology was deemed valid for statistical modelling in the next steps.

### Modelling inter-step interval variability

**Fig 6:**
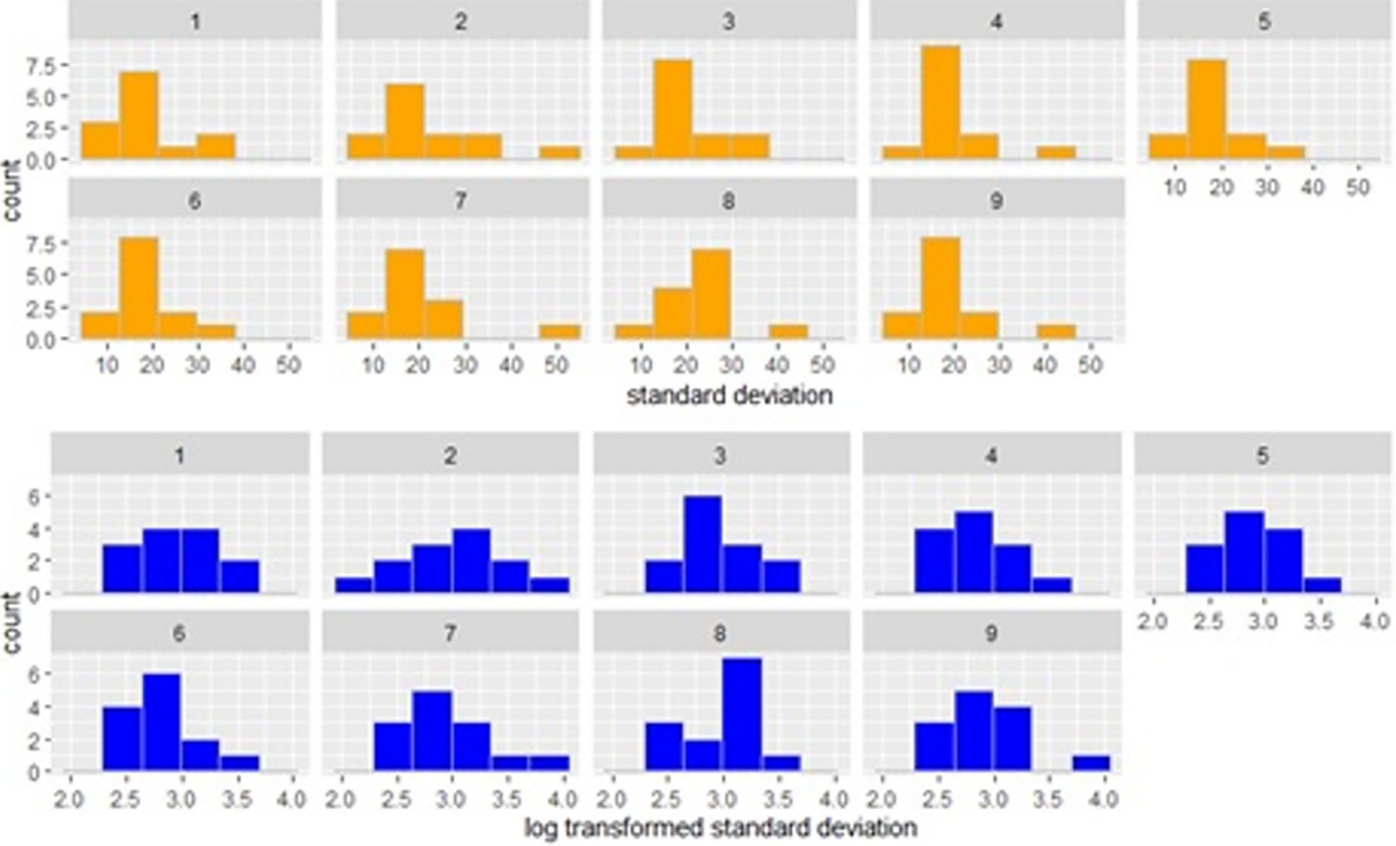
Standard deviation of inter-step-interval from all participants for a specific stimuli and tempi in each window, in original scale (top) and log-transformed (bottom).

The inter-step-interval variability was computed in terms of the standard deviation of the inter-step-interval per window for participant and per trial before moving to model fitting. Fig 6 shows that the data was right-skewed. Therefore, the data was log-transformed (bottom panel) and used in the modelling steps described below.

#### Linear mixed model (LMM)

The log standard deviation changes over the window linearly. For this reason, the log standard deviation was modelled as a linear function of the ordinal variable Window_*k*_. Additionally, for software convenience (related to the convergence of the model), Window_*k*_ was rescaled by dividing it by 10. Since it was of interest to investigate the effect of the locked/unlocked windows and the effect of tempi, the model includes the interaction of these two variables and the two-way interactions of each of them with the other covariates. All main effect of each covariate was also included.

In modelling, model reduction was done on the covariance structure (i.e., random effects) and the mean structure (i.e., covariates). Note that only the interaction effect was reduced while the main effect of each covariate remained in the model regardless of the insignificance. Eventually, the final model only included random intercept and covariates shown in Table 2.

**Table 2:** Parameter estimate of Linear mixed model.

Parameter	Estimate	Std. error	t	p-value
(Intercept)	2.9725	0.0726	40.964	0.000
Tempi: n12	0.1601	0.0378	4.241	0.000
Tempi: n4	0.0125	0.0377	0.333	0.739
Tempi: n8	0.1276	0.0386	3.305	0.001
Tempi: p12	-0.1970	0.0425	-4.637	0.000
Tempi: p4	-0.0562	0.0399	-1.406	0.160
Tempi: p8	-0.1241	0.0431	-2.876	0.004
Stimuli: music	0.2254	0.0383	5.888	0.000
Window_lab_resc	0.0000	0.0283	-0.001	0.999
Lock_lab: unlocked	0.0594	0.0479	1.240	0.215
Tempi: n12 * Stimuli: music	-0.1945	0.0551	-3.533	0.000
Tempi: n4 * Stimuli: music	-0.0200	0.0526	-0.380	0.704
Tempi: n8 * Stimuli: music	-0.1196	0.0562	-2.128	0.034
Tempi: p12 * Stimuli: music	0.0016	0.0542	0.030	0.976
Tempi: p4 * Stimulimusic	-0.1282	0.0558	-2.296	0.022
Tempi: p8 * Stimulimusic	-0.0432	0.0562	-0.769	0.442
Tempi: n12 * Lock_lab: unlocked	0.0518	0.0662	0.783	0.434
Tempi: n4 * Lock_lab: unlocked	0.0162	0.0647	0.250	0.803
Tempi: n8 * Lock_lab: unlocked	-0.0304	0.0699	-0.436	0.663
Tempi: p12 * Lock_lab: unlocked	0.2790	0.0619	4.506	0.000
Tempi: p4 * Lock_lab: unlocked	0.0703	0.0658	1.069	0.285
Tempi: p8 * Lock_lab: unlocked	0.2064	0.0656	3.147	0.002

Based on Table 2, the clinical research questions could be answered, a short summary of such answers is listed below. Note that the answer to specific clinical research questions is not in the scope of the current work.

- There was a significant difference in inter-step interval standard deviation between locked and unlocked windows within Tempi: 0.
- There was a significant effect of Tempi on inter-step interval standard deviation, and the effect was different between the locked and unlocked labels for the two stimuli.

## Discussion

We proposed a methodology to quantify steps-to-beat coupling dynamics of participants over time. This is relevant given the individual differences present during such tasks over time, and more so in neurological populations.

Methodologically, we have quantified the phase in which the subjects were locking or unlocking their step to the beat of stimuli with the proposed method. This approach is different than other studies applying methodologies deriving features of the complete time series [28,29], or specific to gait datasets, features extracted by the means segmentation and feature extraction derived from video recordings of whole body silhouettes [30,31]. Here, the approach we selected was a window partitioning approach -similar to the window segmentation [32]—allowing us to quantify the phase over time for each trial by deriving features in each window. The features could be any measurements that tell us the characteristics of the window. Here, we have derived the slope and the circular statistic in each window to represent the trend and the fluctuation, respectively. These features have been evaluated separately and then combined with a simple decision rule. The features were able to distinguish between the locked and the unlocked window.

The window length of 20 seconds was opted to partition the time series for the method to be more flexible in capturing the dynamic of the synchronisation over time. We do however acknowledge the limitation of this choice, as this may compromise the number of observations of step per window. In this study, given that the observations where of gait data, sufficient number of steps were present within each time window for the application of the circular uniformity and slope tests.

After applying our proposed method and having the label of locking/unlocking in each window (by the combined methodology), the inter-step interval variability has been modelled by an LMM as a function of the locking and unlocking phase, tempi, stimuli, and time represented by the window. This methodology allows us to answer the clinical research question—although out of scope of this work—about the differences in inter-step interval variability between the locking and unlocking phase across groups, tempi and stimuli. It thus provides a more fine-grained understanding on the dynamics of the coupling, which could lead to personalised use of rhythm-based interventions in neurological populations [33].

Regarding the final choice to model the inter-step-interval variability, besides LMM, one can also consider using Generalized Linear Mixed Model (GLMM) or Generalized Estimating Equation (GEE) [34]. These models take into consideration that the observation follows a non-Gaussian distribution. In this study, the response variable was the standard deviation of the inter-step interval. Since it can take value from zero to infinity and has a right-skewed distribution, gamma distribution can be used in GLMM and GEE with log link function. The choice of the model for future analysis depends on the interpretation of interest, as well as the software availabilities and capabilities. In general, for population average interpretation, GEE is practically less software-intensive than GLMM. GLMM requires additional computation to obtain the marginal estimates. On the other hand, GLMM also provides subject-specific interpretation, which can be used to investigate each individual further, e.g., by detecting a subject with an extreme pattern. While the LMM (as was applied here) can also be used for marginal and subject-specific interpretation.

The methodology proposed in this work complements existing metrics of auditory-motor synchronisation which primarily measures synchronisation precision and accuracy [4,35]. While the latter metrics remain crucial for capturing important changes across trials (especially when complemented with the gait spatiotemporal parameters) as shown by studies on Parkinson’s [1,36], multiple sclerosis [3,12,14,37,38] and stroke, our approach offers a more fine-grained analysis. It allows us to quantify changes in dynamics, distinguishing between periods of locked (well-synchronised), and unlocked (poorly synchronised) states. This approach has the potential to enable continuous monitoring and classification. A future step could involve the modification of these dynamics through biofeedback, using auditory signals to adjust behaviour according to clinical needs. These auditory-biofeedback strategies have already been applied in the field of sport science to reduce injuries or enhance performance [39,40] and can now complement personalised music therapy or personalised auditory feedback in rehabilitation [33,41].

The method holds broad potential and warrants future investigations, as it could be applied to quantify auditory-motor dynamics not only in neurological populations with motor impairments, but also in those with cognitive or attentional deficits. For example, these investigations can be extended into the field of paediatric rehabilitation for children with attentional deficits, such as ADHD, autism and developmental coordination disorder and other related pathologies, as auditory-motor coupling strategies have demonstrated effects in these populations [15,42–44]. Given the positive attitude towards technology in healthcare across continents [45–48], this approach has the potential to be effectively integrated into clinical practice.

The sample size justification in this study is inherently tied to the proposed methodology. The proposed methodology can be impacted by the presence of different gait patterns or responses on the rPA, rather than sample size. This is because the classification takes as input the number of steps per defined window and accordingly classifies the window to ‘locked’ or ‘unlocked’ based on the set of pre-defined features. In this context, the important factor of the methodology is the number of observations available per time-window rather than number of participants included in the sample. With our sample, we can confirm that first, the majority of the included patients were diagnosed with cerebellar stroke, and had similar gait characteristics (SARA gait sub-score, median 1) as seen in the Table 1. Second, in the included sample, each time-window contained a median of 33 steps, and was sufficient for the classification to be accurate and precise, as indicated by the results of the ground truth verification. Thus our proposed classification method performs very well and is feasible to apply in both populations.

However, when testing other neurological populations with varied gait patterns, the window length may have to be adapted in order to ensure appropriate number of observations as well as considering the adaptation features for the evaluation of rPA in each window. Classification approaches in other fields such as neuroimaging [49] and biomechanics [50] rely on appropriate multi-feature selection to enhance the accuracy and overall success of the classification algorithms. Future work is thus warranted to identify additional relevant features that could increase the accuracy of time-window labelling within our proposed classification algorithm.

Noteworthy, is that the scope of this study was the proposed methodological framework. Given its successful implementation, future work can apply this to investigate relevant clinical research questions, taking into account clinical factors (such as medication use, motor impairment, etc.). Moving forward, sample size calculations would be necessary to determine statistical power when modelling the data after its classification. This will ensure sensitivity to infer the effects of interest, providing robust conclusions to be drawn in response to the clinical questions.

## Conclusion

Our proposed classification methodology demonstrated high accuracy, precision and recall in classifying and quantifying the dynamics of step-to-beat coupling over time. The method has the potential to contribute to the monitoring and assessment of individual differences step-to-beat dynamics over time in adult populations, both with and without neurological impairments. Future studies are warranted to validate this approach in larger cohorts across a range of neurological conditions. Ultimately, this methodology can facilitate the development of assistive technologies for clinicians, enabling real-time interventions to adjust the auditory-motor paradigm when suboptimal dynamics are detected, thereby fostering more personalised and effective approach to rehabilitation.
